# Cross-sectional study on the relationship between physiologic and mental health status and hypertension risk among oil workers in Xinjiang

**DOI:** 10.3389/fcvm.2025.1503662

**Published:** 2025-09-26

**Authors:** Zhixin Song, Xiaofan Ma, Xue Li, Jiwen Liu

**Affiliations:** School of Public Health, Xinjiang Medical University, Urumqi, China

**Keywords:** hypertension, mental health, physiologic health, oil workers, TG

## Abstract

**Background:**

Hypertension is a major global risk factor for cardiovascular disease. According to the 2023 Global Hypertension Report, its prevalence among adults aged 30–79 years is 33%, closely mirrored in China at 31.6% (2023 Cardiovascular Health and Diseases in China Report). The etiology of hypertension is multifactorial, involving psychological stress (impacting neuroendocrine and inflammatory pathways) and abnormal lipid metabolism. However, the association between mental health and biomarkers in occupational groups, particularly oil workers, remains unclear.

**Purpose:**

This study using a cross-sectional design to deeply analyze the associations between mental health, physiologic health and hypertension, and to explore the influencing factors of hypertension. It aims to provide a scientific basis for the prevention and treatment of hypertension among oil workers, and also to offer theoretical support for further formulating effective measures to improve the physical and mental health levels of oil workers.

**Materials and methods:**

A cluster sampling method was used to recruit 6,083 active oil workers from the Occupational Health Examination Department of Karamay Central Hospital in Xinjiang, China, from September 2018 to September 2019. Physiologic health status was randomly assessed in 20% of the participants to analyze the dose-response relationship between mental health, physiologic health, and hypertension. Based on the restricted cubic spline model, we explored the dose-response relationship between biochemical indicators and hypertension under different levels of mental health.

**Results:**

The results showed that the prevalence of hypertension among the study population was 18.4%, with significant differences observed across various demographic factors (gender, age, education, marital status, monthly income, work experience, job type, professional title, shift work, cigarette smoking, and alcohol consumption; *P* < 0.05). There was also a statistical difference in the prevalence of hypertension among different mental health states (*P* < 0.05), with a higher prevalence (25.5%) among those with poor mental health. Triglycerides [TG] (OR: 2.11; 95% CI: 1.82–2.45), lactate dehydrogenase [LDH] (OR: 1.02; 95% CI: 1.01–1.02) and low-density lipoprotein-cholesterol [LDL-C] (OR: 1.95; 95% CI: 1.65–2.30) emerged as risk factors for hypertension. After adjusting for confounding factors, TG, LDH, and LDL-C remained significant risk factors, with TG abnormalities conferring a 1.71-fold higher risk of hypertension (OR: 1.71; 95% CI: 1.43–2.05), LDH abnormalities conferring a 1.01-fold higher risk (OR: 1.01; 95% CI: 1.01–1.02), and LDL abnormalities conferring a 1.32-fold higher risk (OR: 1.32; 95% CI: 1.06–1.64). In the group with poor mental health, before adjusting for confounders, elevated levels of TG (OR: 3.08; 95% CI: 1.92–4.96), LDH (OR: 1.02; 95% CI: 1.01–1.03), and LDL-C (OR: 2.26; 95%CI: 1.46–3.51) all increased the risk of hypertension. After adjustment, TG (OR: 4.17; 95% CI: 1.86–9.31) and LDH (OR: 1.02; 95% CI: 1.01–1.04) remained significant risk factors. A linear dose-response relationship was noted between TG and LDH levels, and hypertension risk (TG: *P* for trend < 0.001, *P* for non-linearity = 0.056; LDH: *P* for trend = 0.008, *P* for non-linearity = 0.114).

**Conclusion:**

In conclusion, a dose-response relationship existed between mental health, TG and LDH levels, and hypertension risk. Attention should be paid to the mental health of oil workers and effective measures should be taken to alleviate mental health issues. Regular monitoring of blood lipid levels is crucial to reduce the risk of hypertension.

## 1 Introduction

Hypertension, as the most prevalent cardiovascular disease, poses a significant risk for ischemic and hemorrhagic strokes and coronary artery disease (1). It is estimated that approximately 9.4 million deaths can be attributed to hypertension-related risk factors annually (1). According to the World Health Organization, the global population of individuals with hypertension (defined as a systolic blood pressure ≥140 mmHg, a diastolic blood pressure ≥90 mmHg, or individuals currently taking an antihypertensive medication) has doubled over the past 3 decades, rising from 650 million in 1990 to 1.3 billion in 2019. Among adults 30–79 years of age in 2019, there were approximately 99 million hypertensives in the Eastern Mediterranean region, 346 million in the Western Pacific, 106 million in Africa, 197 million in the Americas, 230 million in Europe, and 294 million in Southeast Asia. According to the China Cardiovascular Health and Disease Report 2023, the prevalence of hypertension among Chinese adults has reached 31.6%, affecting approximately 245 million individuals, while 43.1% of the population (estimated at 435 million) exhibit high-normal blood pressure. Hypertension remains the most prevalent chronic condition in Xinjiang, with the Chinese Hypertension Guidelines (2023 Revision) reporting a regional prevalence of 30.5%, significantly higher than the national average of 27.5%. Hypertension affects one-third of adults worldwide and is a common and life-threatening condition that can lead to strokes, myocardial infarctions, heart failure, kidney damage, and numerous other health issues (2). The epidemiology of hypertension is influenced by multiple determinants, encompassing both non-modifiable factors (e.g., age, genetic predisposition, and ethnicity) and modifiable risk factors (e.g., high-sodium diet, obesity, physical inactivity, tobacco use, excessive alcohol consumption, and psychological stress).

With the transformation of healthcare models, mental health has garnered increasing attention. It has been reported that approximately 450 million individuals worldwide have mental disorders, with at least one-fourth of households affected by psychological and behavioral disorders (3). The economic burden of mental disorders was approximately USD $2.5 trillion in 2010 and is projected to increase to USD $6 trillion by 2030, accounting for 3%–4% of the gross national product of developed countries (3). Currently, the potential link between mental health and cardiovascular diseases (CVDs) is primarily studied from a behavioral and biological mechanism perspective. Mental illnesses such as depression, bipolar disorder, and anxiety are associated with poor adherence to various healthy behaviors in daily life, including physical activity, cigarette smoking, and compliance with cardiovascular medications. These factors mediate the relationship between mental illness and adverse cardiovascular outcomes to some extent (4). With respect to biological mechanisms, growing evidence suggests that mental illnesses are associated with inflammation, predisposing individuals to autonomic nervous system dysfunction, impaired coronary flow reserve, and increased risk of myocardial ischemia. Furthermore, bidirectional influences and feedback loops may exist, linking behavioral and biological factors, thereby significantly elevating disease risk (5). Multiple studies have reported a connection between mental health and CVDs, yet consensus is elusive (6–8). A previous study has shown that mental health is closely related to increased blood pressure variability among young and middle-aged adults (9). An analysis of data from the Behavioral Risk Factor Surveillance System between 2017 and 2020 involving 593,616 young adults (18–49 years of age) showed that individuals with depression had a higher risk of cardiovascular diseases compared to individuals without depression (10). However, some studies have failed to detect a link between hypertension and mental illnesses, such as depression. Additionally, dyslipidemia is prevalent among patients with hypertension, diabetes, and metabolic syndrome (11), and elevated levels of serum total cholesterol (TC), low-density lipoprotein-cholesterol (LDL-C), and non-high-density lipoprotein-cholesterol (HDL-C) are associated with an increased risk of hypertension (12).

The oilfields in Xinjiang, China, are mostly located in the desolate Gobi Desert, where the summers are scorching hot and the winters are bitterly cold. Oil workers often work for >20 days in a row, far from their families and with no recreational activities. These unique working conditions pose significant challenges to the physical and mental health of oil workers. There are numerous reports on the relationship between mental health and hypertension among oil workers in Xinjiang but few studies have analyzed the interplay between physiologic health, mental health, and hypertension in this population (13–16). Therefore, we conducted a comprehensive survey of the physiologic and mental health status, and prevalence of hypertension among oil workers in Xinjiang. We further delved into the risk factors influencing hypertension and explored the dose-response relationship between biochemical indicators and hypertension as a function of mental health severity using a restricted cubic spline model. This study aimed to provide a scientific basis for preventing and treating hypertension among oil workers and the theoretical support for developing effective measures to improve overall physical and mental health.

## 2 Materials and methods

### 2.1 Participants

Using a cluster sampling method, 6500 on-duty oil workers in Xinjiang were recruited from the Occupational Health Examination Department of the Karamay Central Hospital in Xinjiang between September 2018 and September 2019 as the study participants. According to the Report on Cardiovascular Health and Diseases in China 2023, the prevalence of hypertension among Chinese adults is 31.6%. Based on this estimated prevalence (*p* = 31.6%), the required sample size was calculated using the following formula: $N=(\frac{Z_{1}-\alpha /2}{d}{)}^{2}\times pq$, *p* = 31.6%, q = 68.4%, d = 0.1 × *p* = 0.0316, α=0.05, $Z_{1}-\alpha /2=1.96$, The minimum required sample size for this study was calculated to be *N* = 831 participants. Ultimately, the study successfully collected 6,083 valid questionnaires, achieving a response rate of 93.58%. The inclusion criteria were as follows: (1) age ≥18 years and work experience ≥1 year; and (2) no psychoactive medication use in the past year. The exclusion criteria were as follows: (1) a history of mental illness or family history of mental illness; and (2) unwillingness to participate in the survey. The study plan was approved by the Ethics Committee of Xinjiang Medical University, and all of the participants provided their voluntarily written informed consent before the investigation.

From a total of 6,083 study subjects collected, 1,122 had hypertension (non-hypertensive: *n* = 4,961, hypertensive: *n* = 1,122). Using the random number table method, 50% of the hypertensive patients (*n* = 561) were randomly selected as the case group. Subsequently, from the non-hypertensive subjects, individuals were randomly selected using the same method at a 1:1.5 ratio to form the control group (*n* = 841), to compare the physiological health status between hypertensive and non-hypertensive subjects (Case group: *n* = 561, Control group: *n* = 841). Inclusion Criteria: (1) Age ≥18 years and working tenure ≥1 year; (2) No use of psychotropic medications within the past year. Exclusion Criteria: (1) Personal or family history of mental illness; (2) Those who refused to participate in the survey. The diagnostic criteria for hypertension are detailed in Section 2.2.2 of the Methods. Based on physical examination items [Audiometry, Electrocardiogram (ECG), Chest x-ray, Abdominal ultrasonography] and biochemical indicators (Total Cholesterol, Triglycerides, High-Density Lipoprotein (HDL), Low-Density Lipoprotein (LDL), Total Bilirubin, Direct Bilirubin, Indirect Bilirubin, Alanine Aminotransferase (ALT), Aspartate Aminotransferase (AST), ALT/AST ratio, Lactate Dehydrogenase (LDH), Blood Urea Nitrogen (BUN), BUN/Creatinine ratio, Creatine Kinase (CK), Uric Acid, Total Protein, Albumin, Globulin, Albumin/Globulin ratio, Blood Glucose), subjects with incomplete physiological data were excluded. Consequently, a total of 1,096 subjects (Case group: *n* = 434, Control group: *n* = 662) met the requirements for the physiological health component of this study.

### 2.2 Methods

#### 2.2.1 Sociodemographic characteristics

We administered a self-designed 《 Xinjiang Oilfield Workers Health Survey Questionnaire 》to assess participants’ health status. The questionnaire consisted of two main components: (1) Sociodemographic characteristics: including sex, age, educational level, marital status, monthly income, working years, type of work, professional title, and shift work; (2) Mental Health Status: using the Symptom Checklist-90 (SCL-90) to measure mental health status.

#### 2.2.2 Mental health status

The Symptom Checklist 90 (SCL-90), developed by Derogatis in 1975 (17), was used to assess mental health. The Chinese version of the scale has been verified, with validity coefficients for each symptom ranging from 0.77–0.99 (18). The Chinese version of SCL-90 includes 10 factors (somatization, compulsion, interpersonal sensitivity, depression, anxiety, hostility, phobia, paranoia, psychoticism, and others), totaling 90 items. A 5-point scoring system was adopted, as follows: 1 = none; 2 = mild; 3 = moderate; 4 = severe; and 5 = very severe. The total and factor scores were utilized as indicators for evaluating the mental health status. Higher total and factor scores were associated with poorer mental health status. The criteria for determining mental disorders were as follows: total score ≥160 points; or a score >2 points for any factor.

#### 2.2.3 Hypertension determination

##### 2.2.3.1 Blood pressure measurement

The blood pressure of respondents was measured by professional medical staff from the Occupational Disease Physical Examination Department of the Central Hospital of Karamay City (Xinjiang, China) using a calibrated TM-2656VP electronic sphygmomanometer (A&D, Japan). The respondents were required to avoid consuming stimulating beverages for 30 min and rest quietly for 15–30 min in a quiet environment before blood pressure measurement. During the blood pressure measurement, a sitting position was assumed. The bare arm was inserted into the measuring band and the arm was kept at the same level as the heart with the palm facing upward. The blood pressure was measured two times and the average value was used. If the difference between the two measurements was >5 mmHg, the respondents rested for 5 min and the blood pressure was measured again. The average of the three measurements was taken as the final blood pressure.

##### 2.2.3.2 Criteria for hypertension diagnosis

Hypertension was diagnosed according to the diagnostic criteria provided in the 2018 revised edition of *Guidelines for the Prevention and Treatment of Primary Hypertension in China* (19), as follows: hypertension, diastolic blood pressure ≥90 mmHg and/or systolic blood pressure ≥140 mmHg; and isolated systolic hypertension, diastolic blood pressure <90 mmHg and systolic blood pressure ≥140 mmHg; Individuals who were diagnosed with hypertension previously and the blood pressure is currently well-controlled by medication are still defined as having hypertension.

#### 2.2.4 Determination of biochemical indicators

All research subjects had 5 ml of venous blood drawn and collected in an EDTA anticoagulant tube by professional medical staff in the fasting state in the morning. Biochemical indicator tests were conducted by the hospital Laboratory Department. The tested indicators included TG, TC, HDL-C, VLDL-C, LDH, FPG, TBIL, DBIL, IBIL, ALT, AST, BUN, creatine kinase (CK), S/L ratio, BUN/Cr, TP, ALB, G, and A/G ratio.

The normal ranges for the tested indicators in the serum are as follows, according to the *Guidelines for the Prevention and Control of Dyslipidemia in Chinese Adults* (20): TG:0.56–1.7 mmol/L; TC: 2.84–5.68 mmol/L; HDL-C:1.14–1.91 mmol/L; LDL-C:1.1–3.4 mmol/L; LDH:97–270 U/L; FPG: 3.89–6.1 mmol/L; TBIL:1.7–17.1 μmol/L; DBIL:0–6.0 μmol/L; IBIL:0.2–0.8 mg/L; ALT: 0–40 U/L; AST: 0–45 U/L; BUN: 2.5–7.1 mmol/L; CK: 0–200 U/L; S/L: 0.8–1.5; BUN/Cr:12–20:1; TP: 60–85 g/L; ALB: 35–51 g/L; G: 20–35 g/L; A/G: 1.5–2.5:1.

#### 2.2.5 Measurement and calculation of BMI

Measurements were obtained from all research subjects in a physical examination room at the Medical Examination Center. The research subjects removed shoes, coats, bags, and other items, stood upright on both legs, and looked straight ahead while on an electronic height and weight scale. The body mass index (BMI) was calculated as follows: body weight (kg)/height² (m²). According to the *Guidelines for the Prevention and Control of Overweight and Obesity in Chinese Adults* (21), BMI categories are as follows: underweight, <18.5 kg/m²; normal weight, between 18.5 kg/m² and 24 kg/m²; overweight, between 24.0 kg/m² and 28.0 kg/m²; and obese, ≥28.0 kg/m².

#### 2.2.6 Hearing test

Hearing was tested using an air conduction audiometer (AD226, Interacoustics A/S, Audiometer Alle 1,5500 Middelfart, DENMARK). The research subject entered the pure tone audiometry room, sat on a chair, and wore a blue headset on the left ear and a red headset on the right ear. Whenever the research subject heard a sound, regardless of the volume or which ear the sound was heard in, a button was pressed. Based on the principle of starting the test from the ear with better hearing, the tone began at 500 Hz and decreased by 10 dB until the research subject no longer responded. Then, the tone increased by 5 dB. Each frequency was measured three times. If the response occurred more than twice, the frequency was confirmed as the auditory threshold. Measurements were conducted at frequencies of 500, 1,000, 2,000, 3,000, 4,000, and 6,000 Hz, and the physician made a diagnosis based on the responses of the research subject.

#### 2.2.7 Electrocardiogram measurement

A 12-lead electrocardiogram (Philips PageWriter TC30) was performed on all research subjects with relaxed limbs in a warm room. The research subject lay flat on the examination bed, the skin of the chest and limbs was exposed, and after disinfection with 75% alcohol, chest and limb leads were applied. The electrocardiogram results were recorded and a diagnosis was made.

#### 2.2.8 Pulmonary examination

A Bright Speed Elite 16-slice spiral CT machine (GE, Boston, Massachusetts, USA) was used. The research subject stood at the designated position with the front of the chest closely attached to the cassette and hands spread apart under the guidance of the physician. The research subject inhaled and exhaled as instructed by the physician. The examination focused on lung, pleura, and heart lesions. The radiologist made a diagnosis.

#### 2.2.9 Abdominal color Doppler ultrasonography

Abdominal color Doppler ultrasonography was performed by professional physicians in the Physical Examination Department using a Philips Affiniti 30 machine (Philips, Amsterdam, Netherlands). Prior to testing, the subjects were asked to fast and have a full bladder. The subjects lay flat on the examination bed, as instructed by the physician, relaxed the abdomen a B-ultrasound was performed, and a diagnosis was made. The examination mainly focused on the liver, gallbladder, spleen, and both kidneys.

### 2.3 Quality control

1. Prior to the investigation, contact was made with the surveyed units or the supervisory departments of the surveyed units to explain the purpose, significance, content, and specific measures of the survey, and to seek close collaboration to minimize survey bias. With the consent of the surveyed units, a dedicated person from the surveyed units was assigned to coordinate the survey. Before the formal investigation, training was provided to the randomly selected subjects from each unit regarding the purpose and significance of the survey, specific measures, and relevant issues requiring attention.
2. Surveyors were uniformly trained and the standards and filling instructions for each item of the questionnaire were specified. The survey language was standardized. Questionnaires were distributed on the day of the respondents’ physical examination. The surveyors clarified the purpose and significance of the survey and after obtaining informed consent from the respondents, the questionnaires were distributed. The completed questionnaires were collected on-site.
3. Immediately after the questionnaires were collected, the surveyors organized a review of the data for that day. The surveyors were uniformly numbered and corresponding records were made. Incomplete and poor-quality questionnaires were eliminated. Data were entered into the Epidata database using a double-entry system and 20% of the questionnaires were randomly selected for verification. Only the data from the last collection of the same survey were entered into the database.

### 2.4 Statistical analysis

The data were input into the Epidata 3.0 database. Statistical analysis was performed using SPSS 22.0 software. Quantitative data conforming to a normal distribution and homogeneity of variance were described by appropriate measures. A two independent samples t-test was used for comparing the means of two groups. One-way ANOVA was utilized for comparing the means of three or more groups. Data not obeying a normal distribution were statistically described by the median and interquartile range, and a rank-sum test or ANOVA was used for statistical analysis. Categorical data were described by frequency and constituent ratio, and the chi-square test was used for comparing rates. If the theoretical frequency was <5, Fisher's exact probability method was used. Variables with statistical significance in univariate analysis were incorporated into a binary logistic regression model to identify independent risk factors for hypertension. The dose-response relationship between biochemical indicators and hypertension was analyzed using a restricted cubic spline combined with a logistic regression analysis model. The RCS analysis was conducted using the rms package in R language (version 4.3.2), with a restricted cubic spline curve (RCS) analysis performed using five knots (the 5th, 35th, 50th, 65th, and 95th percentiles). The significance level was set at α = 0.05.

## 3 Results

### 3.1 Basic situation

Among the 6,083 research subjects in this survey, the prevalence of hypertension was 18.4%. The study population comprised 3,956 males and 2,127 females, with ages ranging from 19 to 60 years (41.49 ± 8.40). There were statistically significant differences in the prevalence of hypertension based on gender, age, level of education, marital status, monthly income, years of employment, job type, professional title, shift worked, cigarette smoking, and alcohol consumption (*P* < 0.05) Table 1. The demographic characteristics of cases and controls in the subset population are summarized in Table 2.

**Table 1:** Comparison of hypertension prevalence among petroleum workers with different demographic characteristics.

Variables	Group	*n*	Non-hypertensive group	Hypertension group	*χ* ^2^	*P*
			*N* (%)	*N* (%)		
Sex	Male	3,956	3,031 (76.6)	925 (23.4)	183.345	<0.001
	Female	2,127	1,930 (90.7)	197 (9.3)		
Age group, years	<30	822	781 (95.0)	41 (5.0)	392.731	<0.001
	30-	2,602	2,302 (88.5)	300 (11.5)		
	≥45	2,659	1,878 (70.6)	781 (29.4)		
Educational level	Below junior college	2,507	1,847 (73.7)	660 (26.3)	176.1	<0.001
	Junior college or above	3,576	3,114 (87.1)	462 (12.9)		
Marital status	Single	1,070	913 (85.3)	157 (14.7)	12.28	<0.001
	Married	5,013	4,048 (80.8)	965 (19.2)		
Monthly income, CNY/Yuan	<5,000	394	305 (77.4)	89 (22.6)	7.544	0.023
	5,000-	5,231	4,268 (81.6)	963 (18.4)		
	≥8,000	458	388 (84.7)	70 (15.3)		
Working years	<10	1,652	1,556 (94.2)	96 (5.8)	465.201	<0.001
	10∼	2,051	1,769 (86.3)	282 (13.7)		
	≥20	2,380	1,636 (68.7)	744 (31.3)		
Type of work	Underground work	654	531 (81.2)	123 (18.8)	35.22	<0.001
	logging	500	363 (72.6)	137 (27.4)		
	Oil transportation	1,540	1,287 (83.6)	253 (16.4)		
	Extract oil	2,878	2,345 (81.5)	533 (18.5)		
	Refiner	511	435 (85.1)	76 (14.9)		
Professional title	none	1,296	1,054 (81.3)	242 (18.7)	64.771	<0.001
	Junior title	707	631 (89.3)	76 (10.7)		
	Intermediate title	1,439	1,223 (85.0)	216 (15.0)		
	Senior title	2,641	2,053 (77.7)	588 (22.3)		
Shift	Fixed day shift	2,130	1,783 (83.7)	347 (16.3)	10.107	0.001
	shift	3,953	3,178 (80.4)	775 (19.6)		
BMI	<18.5	123	113 (91.9)	10 (8.1)	419.704	<0.001
	18.5–23.9	2,439	2,219 (91.0)	220 (9.0)		
	24.0–27.9	2,417	1,938 (80.2)	479 (19.8)		
	≥28.0	1,104	691 (62.6)	413(37.4)		
Total		6,083	4,961 (81.6)	1,122 (18.4)		

**Table 2:** Comparison of hypertension prevalence among petroleum workers with different demographic characteristics(subset).

Variables	Group	*n*	Non-hypertensive group	Hypertension group	*χ* ^2^	*P*
			*N* (%)	*N* (%)		
Sex	Male	580	303 (52.2)	277 (47.8)	34.297	<0.001
	Female	516	359 (69.6)	157 (30.4)		
Age group, years	<30	135	96 (71.1)	39 (28.9)	14.068	0.001
	30-	471	297 (63.1)	174 (36.9)		
	≥45	490	269 (54.9)	221 (45.1)		
Educational level	Below junior college	458	269 (58.7)	189 (41.3)	0.915	0.339
	Junior college or above	638	393 (61.6)	245 (38.2)		
Marital status	Single	192	125 (65.1)	67 (34.9)	2.152	0.142
	Married	904	537 (59.4)	367 (40.6)		
Monthly income CNY/Yuan	<5,000	288	186 (64.6)	102 (35.4)	3.279	0.194
	5,000-	675	401 (59.4)	274 (40.6)		
	≥8,000	133	75 (56.4)	58 (43.6)		
Working years	<10	265	164 (61.9)	101 (38.1)	22.235	<0.001
	10∼	475	252 (53.1)	223 (46.9)		
	≥20	356	246 (69.1)	110 (30.9)		
Type of work	Underground work	125	70 (56.0)	55 (44.0)	62.088	<0.001
	logging	103	82 (79.6)	21 (20.4)		
	Oil transportation	501	284 (56.7)	217 (43.3)		
	Extract oil	223	167 (74.9)	56 (25.1)		
	Refiner	144	59 (41.0)	85 (59.0)		
Professional title	None	198	125 (63.1)	73 (36.9)	14.079	0.003
	Junior title	357	213 (59.7)	144 (40.3)		
	Intermediate title	331	178 (53.8)	153 (46.2)		
	Senior title	210	146 (69.5)	64 (30.5)		
Shift	Fixed day shift	372	252 (67.7)	120 (32.3)	12.686	<0.001
	shift	724	410 (56.6)	314 (43.4)		
BMI	<18.5	30	23 (76.7)	7 (23.3)	116.028	<0.001
	18.5–23.9	469	354 (75.5)	115 (24.5)		
	24.0–27.9	387	215 (55.6)	172 (44.4)		
	≥28.0	210	70 (33.3)	140 (66.7)		
Total		1,096	662 (60.40)	434 (39.60)		

### 3.2 Comparison of SCL-90 scores in hypertensive patients

There was a statistically significant difference in the total SCL-90 score and the factor scores between patients with and without hypertension (*P* < 0.05). The SCL-90 and 10 factor scores in the hypertensive group were higher than the non-hypertensive group. The total SCL-90 score in the hypertensive group (161.17 ± 43.22) was higher than the non-hypertensive group (137.09 ± 40.07) Table 3.

**Table 3:** Comparison of SCL-90 scores between hypertensive and non-hypertensive patients.

Variables	Non-hypertensive group (*n* = 4,961)	Hypertensive group (*n* = 1,122)	*t*	*P*
SCL-90 total score	137.09 ± 40.07	161.17 ± 43.22	−17.077	<0.001
Somatization	1.52 ± 0.50	1.83 ± 0.56	−17.044	<0.001
Compulsive symptoms	1.39 ± 0.47	1.63 ± 0.52	−14.445	<0.001
Interpersonal sensitivity	1.47 ± 0.49	1.72 ± 0.52	−14.532	<0.001
Depression	1.88 ± 0.49	2.14 ± 0.51	−15.390	<0.001
Anxiety	1.83 ± 0.46	2.06 ± 0.48	−14.646	<0.001
Hostility	1.25 ± 0.44	1.61 ± 0.48	−23.189	<0.001
Fear	1.29 ± 0.47	1.61 ± 0.53	−18.808	<0.001
Paranoia	1.53 ± 0.48	1.70 ± 0.48	−10.811	<0.001
Psychosis	1.42 ± 0.46	1.66 ± 0.50	−14.689	<0.001
Another factors	1.28 ± 0.47	1.61 ± 0.50	−19.623	<0.001

### 3.3 Comparison of the prevalence of hypertension as a function of mental health status

The survey results demonstrated that 35.44% (2,157/6,083) of oilfield workers screened positive for mental disorders. There was a statistically significant difference in the prevalence of hypertension in research subjects with different mental health conditions (*P* < 0.05). In the negative mental health symptom group, there were 3,354 non-hypertensive individuals (85.4%) and 573 hypertensive individuals (14.6%). In the mental disorder group, there were 1,607 non-hypertensive individuals (74.5%) and 549 hypertensive individuals (25.5%) Table 4. Hypertension prevalence varied significantly by mental health level in the subset Table 5.

**Table 4:** Comparison of the prevalence of hypertension under different mental health levels.

Variables	*n*	Non-hypertensive group *n* (%)	Hypertensive group *n* (%)	*χ* ^2^	*P*
Mental health	3,927	3,354 (85.4)	573 (14.6)	109.377	<0.001
Mental disorder	2,156	1,607 (74.5)	549 (25.5)		

**Table 5:** Comparison of the prevalence of hypertension under different mental health levels (subset).

Variables	*n*	Non-hypertensive group *n* (%)	Hypertensive group *n* (%)	*χ* ^2^	*P*
Mental health	895	591 (66.0)	304 (34.0)	64.721	<0.001
Mental disorder	201	71 (35.3)	130 (64.7)		

### 3.4 Comparison of physiologic indicators between hypertensive and non-hypertensive patients

Statistically significant differences (*P* < 0.05) were detected with respect to the physiologic indicators between the hypertensive and non-hypertensive groups, except for TBIL, DBIL, IBIL, BUN/Cr, TP, ALB, G, and the A/G ratio. Specifically, in the hypertensive group the following indicators were all higher than the non-hypertensive group: TG, 1.74 (1.12–2.65); TC, 5.03 (4.48–5.74); LDL-C, 3.1 (2.62–3.65); LDH, 181.41 (162.18–209.85); FPG, 5.02 (4.73–5.61), ALT, 23 (16.1–35.26); AST, 19.91 (16.72–25.35); BUN, 5.01 (4.2–5.82); and CPK, 96 (73.7–133.61). The following indicators were lower in the hypertensive group than the non-hypertensive group: HDL-C, 1.22 (1.02–1.42); and the S/L ratio, 0.87 (0.68–1.11) Table 6.

**Table 6:** Analysis of the physical health status of hypertensive and non-hypertensive patients among Xinjiang oil workers.

Variables	Non-hypertensive group (*n* = 662)	Hypertensive group (*n* = 434)	$\chi^{2}$	*P*
TG [M(P25, P75)]	1.08 (0.73,1.58)	1.74 (1.12,2.65)	12.505	<0.001
TC [M(P25, P75)]	4.64 (4.1,5.26)	5.03 (4.48,5.74)	7.321	<0.001
HDL-C [M(P25, P75)]	1.37 (1.17,1.6)	1.22 (1.02,1.42)	−7.726	<0.001
VLDL-C [M(P25, P75)]	2.7 (2.27,3.25)	3.1 (2.62,3.65)	8.262	<0.001
LDH [M(P25, P75)]	169.3 (153,187)	181.41 (162.18,209.85)	7.065	<0.001
FPG [M(P25, P75)]	4.86 (4.59,5.21)	5.02 (4.73,5.61)	5.317	<0.001
TBIL [M(P25, P75)]	11.7 (8.6,16)	11.82 (9.38,16.22)	0.887	0.375
DBIL [M(P25, P75)]	2.04 (1.59,2.75)	2.1 (1.52,2.75)	0.248	0.804
IBIL [M(P25, P75)]	9.57 (6.92,13.28)	9.7 (7.61,13.51)	0.77	0.441
ALT [M(P25, P75)]	17.8 (12.6,26.88)	23 (16.1,35.26)	6.964	<0.001
AST [M(P25, P75)]	18.5 (15.58,22.6)	19.91 (16.72,25.35)	4.974	<0.001
BUN [M(P25, P75)]	4.62 (3.92,5.55)	5.01 (4.2,5.82)	3.947	<0.001
CK [M(P25, P75)]	81.97 (62.15,116)	96 (73.7,133.61)	5.088	<0.001
S/L [M(P25, P75)]	1.03 (0.78,1.33)	0.87 (0.68,1.11)	−6.465	<0.001
BUN/Cr [M(P25, P75)]	0.06 (0.05,0.07)	0.06 (0.05,0.07)	0.509	0.611
TP	75.19 ± 3.82	75.58 ± 4.14	−1.613	0.107
ALB	46.36 ± 2.60	46.40 ± 2.73	−0.227	0.820
G[M(P25, P75)]	28.6 (26.47,31.1)	29.1 (26.61,31.3)	1.361	0.174
A/G[M(P25, P75)]	1.61 (1.46,1.77)	1.61 (1.46,1.77)	−0.807	0.420

### 3.5 Distribution of physiologic abnormalities in hypertensive and non-hypertensive patients

There was a statistically significant difference in the distribution of abnormal hearing, and abdominal ultrasound findings between hypertensive and non-hypertensive patients (*P* < 0.05). Among non-hypertensive patients, the proportions of underweight, normal weight, overweight, and obese subjects were 3.5%, 53.5%, 32.5%, and 10.6% respectively. The proportion of individuals with hearing abnormalities in the non-hypertensive group was lower (25.4%) than the hypertensive group (32.5%). The proportion of individuals with abnormal abdominal ultrasound findings in the hypertensive group was higher (80.6%) than the non-hypertensive group (70.1%) Table 7.

**Table 7:** Distribution of abnormal physiologic health in hypertensive and non-hypertensive workers in the Xinjiang oilfield.

Variables	Group	Non-hypertensive group (*n* = 662)	hypertensive group (*n* = 434)	$\chi^{2}$	*P*
Hearing	Normal	494 (74.6)	293 (67.5)	6.547	0.011
	Abnormal	168 (25.4)	141 (32.5)		
Chest radiograph	Normal	593 (89.6)	388 (89.4)	0.009	0.926
	Abnormal	69 (10.4)	46 (10.6)		
Electrocardiogram	Normal	535 (80.8)	339 (78.1)	1.188	0.276
	Abnormal	127 (19.2)	95 (21.9)		
Lung function	Normal	630 (95.2)	415 (95.6)	0.123	0.726
	Abnormal	32 (4.8)	19 (4.4)		
Abdominal ultrasound	Normal	198 (29.9)	84 (19.4)	15.281	<0.001
	Abnormal	464 (70.1)	350 (80.6)		

### 3.6 Multivariate analysis of risk factors for hypertension

The variables that were significant in the univariate analysis were introduced into the equation for binary logistic regression analysis. The subsample analysis indicated that TG (OR: 2.11; 95% CI: 1.82–2.45), LDH (OR: 1.02; 95% CI: 1.01–1.02), and LDL-C (OR: 1.95; 95% CI: 1.65–2.30) were risk factors for hypertension. After correcting for confounding factors, TG, LDH, and LDL-C remained risk factors for hypertension. Specifically, the risk of developing hypertension for subjects with an abnormal TG, LDH, and LDL-C level was 1.71 (OR: 1.71; 95% CI: 1.43–2.05), 1.01 (OR: 1.01, 95% CI: 1.01–1.02), and 1.32(OR:1.32, 95% CI: 1.06–1.64) that of subjects with a normal TG, LDH, and LDL-C level, respectively Table 8.

**Table 8:** Logistic regression analysis of influencing factors of hypertension.

Variables	Model 1	*P*	Model 2	*P*
	OR (95%CI)		OR (95%CI)	
TG	2.11 (1.82–2.45)	<0.001	1.71 (1.43–2.05)	<0.001
LDH	1.02 (1.01–1.02)	<0.001	1.01 (1.01–1.02^)^	<0.001
LDL-C	1.95 (1.65–2.30)	<0.001	1.32 (1.06–1.64)	<0.050

Model 1 did not adjust for confounding factors. Model 2 for TG was adjusted for sex, age, working years, type of work, professional title, shift, BMI, Mental health, TC, HDL-C, VLDL-C, LDH, FPG, ALT, AST, BUN, CK, S/L, hearing, abdominal ultrasound. Model 2 for LDH was adjusted for, sex, age, working years, type of work, professional title, shift, BMI, mental health, TG, TC, HDL-C, VLDL-C, FPG, ALT, AST, BUN, CK, S/L, hearing, abdominal ultrasound. Model 2 for LDL-C was adjusted for sex, age, working years, type of work, professional title, Shift, BMI, mental health, TG, TC, HDL-C, LDH, FPG, ALT, AST, BUN, CK, S/L, hearing, abdominal ultrasound.

### 3.7 Risk of hypertension associated with TG, LDH, and LDL indices for different mental health states

The results of the stratified analysis based on mental health symptoms revealed that in the negative mental health symptom group, both before and after adjusting for confounding factors, the TG (OR before adjustment: 2.00; 95% CI before adjustment: 1.70–2.33; OR after adjustment: 1.56, 95% CI after adjustment: 1.32–1.85), LDH (OR before adjustment: 1.02; 95% CI before adjustment: 1.01–1.02; OR after adjustment: 1.01, 95% CI after adjustment: 1.01–1.02), and LDL-C level (OR before adjustment: 1.90, 95% CI before adjustment: 1.58–2.29; OR after adjustment: 1.35, 95% CI after adjustment: 1.08–1.69) were risk factors for hypertension. In the positive mental health symptom group, elevated levels of TG (OR: 3.08, 95% CI: 1.92–4.96), LDH (OR: 1.02, 95% CI: 1.01–1.03), and LDL-C (OR: 2.26, 95% CI: 1.46–3.51) increased the risk of hypertension before adjusting for confounding factors. TG (OR: 4.17, 95% CI: 1.86–9.31) and LDH (OR: 1.02, 95% CI: 1.01–1.04) remained as risk factors for hypertension after adjusting for confounding factors Table 9.

**Table 9:** Logistic regression analysis of the association between TG, LDH, LDL-C indices and hypertension under different mental health states.

Variables	Mental health				Mental disorders			
	Model 1	*P*	Model 2	*P*	Model 1	*P*	Model 2	*P*
	OR (95%CI)		OR (95%CI)		OR (95%CI)		OR (95%CI)	
TG	2.00 (1.70–2.33)	<0.001	1.56 (1.32–1.85)	<0.001	3.08 (1.92–4.96)	<0.001	4.17 (1.86–9.31)	<0.001
LDH	1.02 (1.01–1.02)	<0.001	1.01 (1.01–1.02)	<0.001	1.02 (1.01–1.03)	0.028	1.02 (1.01–1.04)	0.017
LDL-C	1.90 (1.58–2.29)	<0.001	1.35 (1.08–1.69)	0.008	2.26 (1.46–3.51)	<0.001	1.28 (0.62–2.63)	0.507

Model 1 did not adjust for confounding factors. Model 2 for TG was adjusted for sex, age, working years, type of work, professional title, shift, BMI,TC,HDL-C,VLDL-C, LDH, FPG, ALT, AST, BUN, CK,S/L, hearing, abdominal ultrasound. Model 2 for LDH was adjusted for, sex, age, working years, type of work, professional title, shift, BMI, TG, TC, HDL-C, VLDL-C, FPG, ALT, AST, BUN, CK, S/L, hearing, abdominal ultrasound. Model 2 for LDL-C was adjusted for sex, age, working years, type of work, professional title, shift, BMI, TG, TC, HDL-C, LDH, FPG, ALT, AST, BUN, CK, S/L, hearing, abdominal ultrasound.

### 3.8 Dose-response relationship between TG, LDH, and LDL-C indices of oil workers and the risk of hypertension

**Figure 1:**
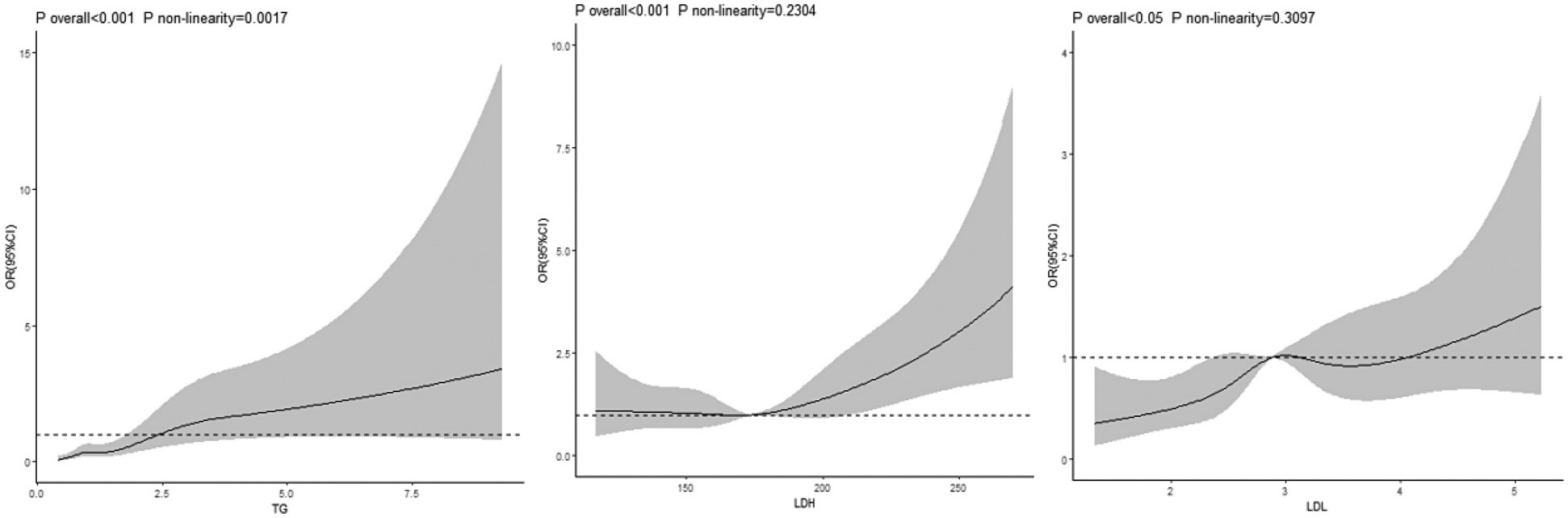
Dose-response relationship between TG, LDH, LDL index and risk of hypertension in petroleum workers.

After adjusting for relevant confounding factors, a non-linear dose-response relationship existed between the TG index and risk of hypertension (*P* for overall trend <0.001, *P* for non-linearity <0.05). A linear dose-response relationship was noted between the LDH and LDL indices and the risk of hypertension (LDH: *P* for overall trend <.001, *P* for non-linearity = 0.230; LDL: *P* for overall trend < 0.05, *P* for non-linearity = 0.309) Figure 1.

### 3.9 Dose-response relationship between TG, LDH, and LDL-C indices and the risk of hypertension under different mental health states

**Figure 2:**
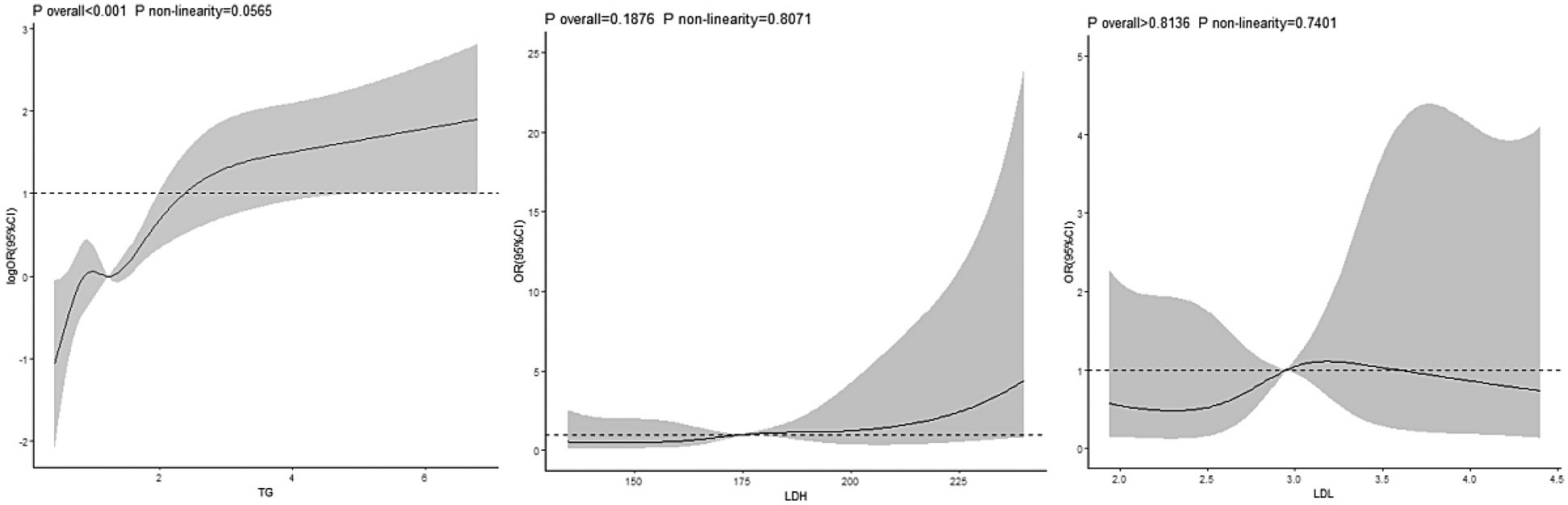
Dose-response relationship between TG, LDH, LDL and hypertension in negative psychological symptoms group.

**Figure 3:**
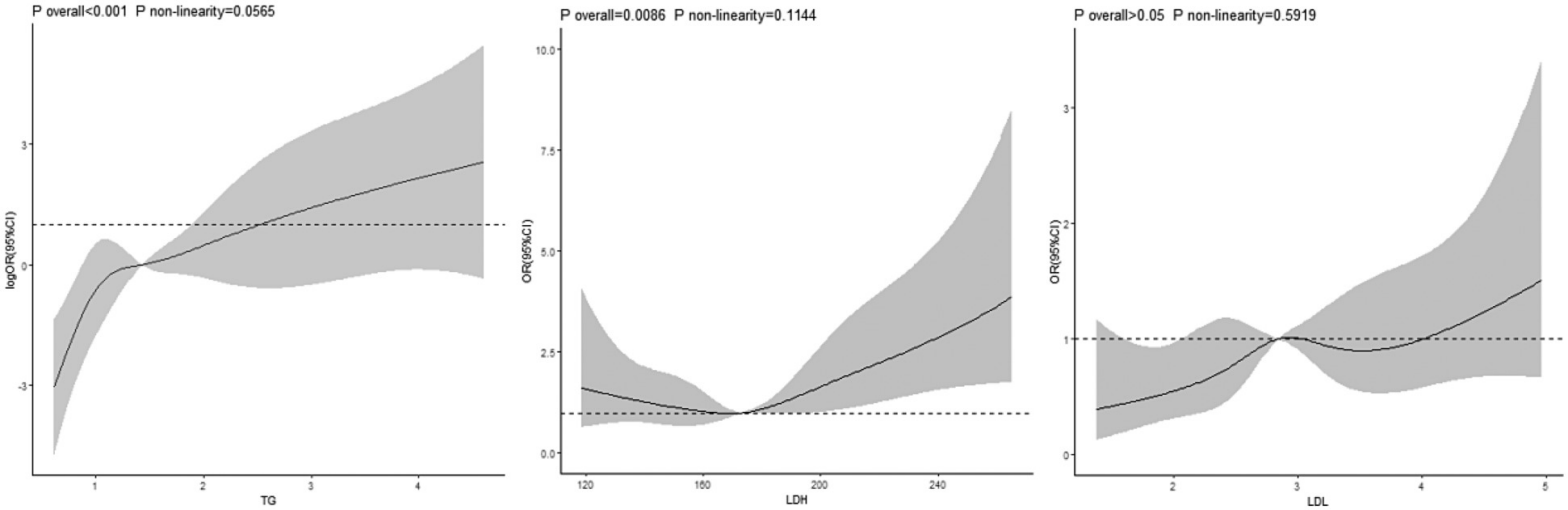
Dose-response relationship between TG, LDH, LDL and hypertension in positive psychological symptoms group.

In the group with negative psychological symptoms, a linear dose-response relationship existed between the TG index and the risk of hypertension (*P* for overall trend <0.001, *P* for non-linearity = 0.056). No dose-response relationship was observed between the LDH and LDL-C indices and the risk of hypertension (LDH: *P* for overall trend >0.05, *P* for non-linearity = .807; LDL-C: *P* for overall trend >0.05, *P* for non-linearity = 0.740) Figure 2. In the group with positive psychological symptoms, there were linear dose-response relationships between the TG and LDH indices and the risk of hypertension (TG: *P* for overall trend <0.001, *P* for non-linearity = 0.056; LDH: *P* for overall trend = 0.008, *P* for non-linearity = 0.114) Figure 3.

## 4 Discussion

Hypertension has been declared a global public health crisis by the World Health Organization (WHO) because of its high prevalence: it affects >1 billion people worldwide and directly causes >10 million deaths each year (22). Consequently, many scientists have focused their efforts on hypertension research in various directions. Studies have indicated that the risk factors for hypertension include age, gender, lifestyle, obesity, and immunity (23). Our study identified a hypertension prevalence of 18.4% among oil workers, significantly lower than the national prevalence of 27.5% reported in the 2023 Chinese hypertension guidelines for adults aged ≥18 years, yet comparable to earlier estimates in this occupational group (24–26). The results of this study indicate that the prevalence of hypertension among male oil workers is higher than that among females, which is inconsistent with the findings of Yang et al. (25) in 2021 but aligns with the conclusions of Zhang et al. (27). Additionally, the 2018 China Chronic Disease and Risk Factors Surveillance (CCDRFS) survey also revealed a higher prevalence of hypertension in males compared to females. Furthermore, previous study have found that the risk of hypertension increases with higher BMI, which is consistent with the conclusions of this study (25). The significantly lower prevalence of hypertension among oil workers compared to the national adult level is primarily due to the fact that the oil industry typically sets medical examination thresholds for blood pressure, cardiopulmonary function, etc. during recruitment, eliminating those with existing hypertension or cardiovascular diseases, resulting in on-the-job workers having a baseline health level superior to that of the general population. Meanwhile, as types of work such as oil workers’ drilling and equipment maintenance require continuous physical labor (with an average daily step count of over 15,000 steps), this helps maintain a body weight/BMI lower than that of sedentary populations (such as urban white-collar workers). In addition, long-term physical labor increases skeletal muscle mass and improves insulin sensitivity, indirectly reducing the risk of metabolic syndrome (one of the core mechanisms of hypertension).

The results of this study indicate that the positive detection rate of mental health symptoms among oil workers is 35.44%, which is lower than the 48.5% prevalence of psychological disorders reported by Li (28) et al. in 2022. This suggests that increased awareness of mental health issues among workers may lead to earlier adoption of self-regulation strategies (such as exercise and social activities) or proactive help-seeking behaviors, thereby preventing the exacerbation of problems. However, due to their unique occupational environment (harsh natural conditions, high-risk operations, shift work, and circadian rhythm disorders) and job characteristics (long-term absence from home with limited social interaction, work-family conflict, and scarcity of cultural and recreational activities), the detection rate of psychological disorders among oil workers remains higher than that of general occupational populations. The results of this study indicate that the prevalence of hypertension is higher in the group with positive mental health symptoms. Research has demonstrated that there is an association between mental health and hypertension; specifically, anxiety disorders and depression are related to hypertension and mental illnesses are associated with increased blood pressure variability in young and middle-aged individuals (29). Hypertension and affective disorders, such as depression, frequently co-occur and it has also been shown that affective disorders are risk factors for many cardiovascular diseases (9). It has been reported that patients with affective disorders have an increased risk of hypertension and CVD risk factors, including hypertension, have also been shown to cause depressive symptoms, such as aging through microvascular brain damage (30). Kwapong et al. (10) consider depression as a non-traditional risk factor for CVDs. The incidence of CVDs in patients with depression was higher than non-depressed patients (OR: 2.32; 95% CI: 2.13–2.51) based on data involving 593,616 young people (18–49 years of age) monitored by the Behavioral Risk Factor Surveillance System from 2017 to 2020. Depression and poor mental health are associated with premature CVDs in young people and the two interact. Maintaining good mental health may help reduce the risk of cardiovascular diseases and improve the problem of poor cardiovascular health in young people. Ang et al. (31) showed that hypertension may be a contributing factor for mental illnesses. Compared to individuals who received hypertension screening and had controlled blood pressure, individuals who did not undergo hypertension screening and did not control blood pressure had a higher risk of anxiety and depression. Respondents who did not take hypertension medications had a lower probability of developing anxiety and depression compared to respondents who took hypertension medications. There is an association between different degrees of hypertension and mental illnesses. There is also evidence suggesting a bidirectional relationship between depression and hypertension, which leads to a reduced quality of life, lower treatment adherence rates, and increased mortality in older adult patients with hypertension (32). Additionally, a non-randomized study indicates that hypertensive patients with depressive symptoms require more antihypertensive drugs to achieve good blood pressure control.

The results of this study indicated that the TG, TC, and LDL-C levels in hypertensive patients are higher than non-hypertensive individuals, while the HDL-C level is lower than non-hypertensive individuals. There were differences in the abnormal incidence of TG, TC, and LDL-C levels between the two groups. Previous studies have reported an interaction between blood lipids and blood pressure, which may be the basis of the common pathophysiologic mechanisms of dyslipidemia and hypertension, such as endothelial dysfunction and reduced arterial elasticity (33). Studies have shown that dyslipidemia is common in patients with hypertension, diabetes, and metabolic syndrome (11). Elevated serum TC, LDL-C, and non-HDL-C levels are all associated with an increased risk of hypertension (12). A study including 403,335 participants from the UK Biobank determined the association between the triglyceride-glucose (TyG) index and the TG-to-HDL-C (TG/HDL-C) ratio, and CVD in the European population. The multivariable-adjusted hazard ratios for total CVD in higher quartiles vs. the lowest quartiles were 1.05, 1.05, and 1.19, respectively, for the TyG index, and 1.07, 1.13, and 1.29, respectively, for the TG/HDL-C ratio. There were significant trends toward an increasing risk of CVD across the TyG index and TG/HDL-C ratio quartiles. Dyslipidemia, type 2 diabetes, and hypertension accounted for 45.8%, 27.0%, and 15.0% of the TyG index association with CVD, respectively, and 40.0%, 11.8%, and 13.3% of the TG/HDL-C ratio association with CVD, respectively, in mediation analyses (34). Furthermore, a population cohort study in middle-aged men without baseline hypertension in Finland revealed that for each standard deviation increase in triglyceride (TG) concentration, the risk of new-onset hypertension increased by 1.6-fold (95% CI: 1.2–2.3), while the HDL-C concentration (OR: 0.7, 95% CI: 0.5–0.9) appeared to have a protective effect (35). Currently, various pathophysiologic mechanisms have been proposed to explain the association between the risk of new-onset hypertension and lipid levels. Dyslipidemia can modify the permeability of cell membranes and induce renal microvascular injury, thereby leading to hypertension. Studies have indicated that patients with dyslipidemia have deficient nitric oxide bioactivity, resulting in reduced vasodilatory capacity and elevated blood pressure (36). Additionally, atherosclerotic lesions caused by endothelial dysfunction are associated with increased arterial stiffness and decreased arterial compliance, thereby contributing to hypertension (37). Some researchers have speculated that hypertriglyceridemia may compromise vascular dilation mechanisms, thereby increasing vascular resistance and resulting in elevated blood pressure. High TG levels lead to the formation of small, dense LDL-C particles, which are particularly prone to oxidation and form oxidized small, dense LDL-C particles. These oxidized small, dense LDL-C particles have been shown to induce endothelial dysfunction by attenuating endothelial nitric oxide synthesis activity (38). Hence, hypertriglyceridemia may impair vascular dilation mechanisms independently of LDL damage. Moreover, lipolysis of TG in adipose tissue has been shown to lead to the production of non-esterified fatty acids, which have been shown to disrupt endothelial function by inhibiting endothelium-dependent hyperpolarization, a potent vasodilator in small resistance arteries in rats and humans (39). Stratified analysis was performed to further investigate the association between blood lipids and hypertension in the mental health symptom-negative and positive groups. The study found that regardless of mental health status, elevated LDH levels are associated with an increased risk of hypertension. As a sensitive marker of cellular damage and hypoxia, elevated LDH may lead to vascular endothelial injury or metabolic disturbances, thereby contributing to a higher risk of hypertension. This conclusion has also been confirmed by study from other scholar (40).

The study found that in the mental health symptom-negative group, elevated LDL was associated with a higher risk of hypertension. However, in the mental health symptom-positive group, adjusted LDL showed no significant association with hypertension, suggesting that mental health status may act as an effect modifier influencing the relationship between LDL and hypertension. In individuals with positive mental health symptoms, chronic psychological stress may activate the hypothalamic-pituitary-adrenal (HPA) axis and sympathetic nervous system, promoting inflammatory responses and insulin resistance, thereby exacerbating metabolic disorders. These pathophysiological changes may partially or completely mask the hypertension-inducing effects of LDL itself, leading to a weakened or even eliminated independent effect of LDL after adjusting for confounders. Additionally, mental health problems may be accompanied by unhealthy behaviors (e.g., irregular diet, lack of physical activity), further confounding the relationship between LDL and hypertension. Clinical implications: Future hypertension risk assessment and intervention strategies should incorporate mental health status, particularly in patients with metabolic abnormalities, requiring comprehensive management of psychological and physiological risk factors. Further research with a larger sample size is needed to examine the interaction between LDL and mental health and clarify the underlying mechanisms.

The results of this study indicate that in the group with positive psychological symptoms, there exists a linear dose-response relationship between TG, the LDH index, and the risk of hypertension (41–43). Previous study have demonstrated that stressful emotions may cause alterations in blood pressure, cholesterol and TG levels, hematocrit, fibrinogen level, and blood fluidity (42). Stress may result in abnormal activation of the sympathetic nervous system, thereby triggering a hormonal cascade that interferes with blood pressure, and increased coagulation and platelet activity. These factors can serve as “triggers” for the occurrence of cerebrovascular events. Epidemiologic studies have repeatedly investigated the association between anxiety, depression, and hypertension, and have found that depression is an important and independent risk factor for hypertension, particularly more pronounced among young individuals (44–46). Depression can reduce heart rate variability and an excessively low heart rate indicates a greater risk of death due to CVDs (44–46). Similarly, poor mental health status may exacerbate inflammatory responses or elevate blood cortisol levels (47).

## 5 Conclusion

This study showed that in addition to demographic characteristics, positive mental health symptoms and abnormal TG and LDL-C levels increase the risk of hypertension in oil workers. The analysis revealed that there is a non-linear dose-response relationship between the TG index and the risk of hypertension, and a linear dose-response relationship between the LDH and LDL-C indices and the risk of hypertension. Furthermore, in the group with positive psychological symptoms, there was also a linear dose-response relationship between the TG and LDH indices and the risk of hypertension. Limitations:The shortcomings of this study were as follows. First, this was a cross-sectional study, making it challenging to infer causal relationships. Second, as a special occupational group, oil workers have a higher incidence of mental health issues compared to the general working population. The results await further validation in more occupational groups. In this study only the SCL-90 measurement tool was used for measurement of mental health levels, which can merely reflect the overall mental health status. In future research, other measurement tools will be used to measure specific mental health indicators, such as anxiety, depression, and emotional disorders, and to further analyze their potential correlations. Finally, while this study diagnosed hypertension based on two to three blood pressure measurements during a single examination—a conventional approach in epidemiological surveys—this methodology differs from clinical diagnostic standards. Future studies should adopt more rigorous clinical protocols to enhance the reliability of findings.

## Data Availability

The raw data supporting the conclusions of this article will be made available by the authors, without undue reservation.
